# The resonant slop machine: public diplomacy and strategic narratives in the age of artificial intelligence

**DOI:** 10.1057/s41254-025-00419-z

**Published:** 2025-12-15

**Authors:** Rhys Crilley, Robert A. Saunders

**Affiliations:** 1 University of Glasgow, Glasgow, UK; 2 Farmingdale State College, Farmingdale, USA

**Keywords:** Strategic narratives, Public diplomacy, Artificial intelligence, Meme culture, Media ecology, Strategic communication

## Abstract

What does the rise of generative Artificial Intelligence and meme-driven political communication mean for public diplomacy and strategic narratives? Using Donald Trump’s AI-generated “King Trump” fighter jet video (posted during the 2025 “No Kings” protests) as a jumping off point, we explore how AI slop and “shitposting” destabilize the communicative foundations of public diplomacy and international order. By building on Miskimmon, O’Loughlin, and Roselle’s foundational framework of strategic narratives, we argue that the proliferation of AI-generated content transforms global media ecologies, erodes epistemic authority, and amplifies performative, right wing populism. We theorise this phenomenon as the resonant slop machine: a global political-socio-cultural-economic-technological assemblage that fuses generative AI, meme culture, and affective politics to produce incoherent yet resonant narratives that shape public diplomacy. By situating Trump’s digital theatrics within broader trends of diplomatic trolling and post-truth politics we highlight the implications for scholars and practitioners seeking to salvage meaningful public diplomacy in a fractured, enshittified information environment.

## Introduction

The scene is set: a second ‘No Kings’ protest in the United States is gathering steam. Act I: We see a US fighter jet with the words ‘King Trump’ embossed in gold capital letters on the underside. In the cockpit, Donald Trump sits behind the controls, dressed in a military flight suit. With a crown upon his head, the president takes to the skies. Kenny Loggins’ song ‘Danger Zone’—famous for its usage in the propaganda-and-popcorn Reagan-era film *Top Gun* (1986) – blasts from the speakers. ACT II: Zooming over an American city, Trump opens the bomb bay doors, and massive brown lumps of shit are deployed on the targets below. We cut to a young activist, walking the streets with a smile on his face. His grin disappears as he is drenched in a brown soup of faeces. Excrement soon inundates throngs of other protesters proceeding through what looks like Times Square.

What is going on? We ask this as we watch the short video, which is generated using artificial intelligence (AI), posted on the pro-Trump X account Xerias, and then mass-distributed on the social media platform Truth Social by the sitting president, Donald Trump himself. This artefact is a politically-inflected manifestation of what has come to be referred to as ‘AI slop’: a comparatively new field of ‘art’ and media which relies on generative AI (genAI) and employs existing content to produce a ‘waste stream’ of ‘lazy, derivative, endlessly recycled outputs from text, image, and avatar models’ (Madsen and Puyt 2025). Given that since the early days of his first administration, any social media posts made by the president are to be considered ‘official statements’ of White House policy (Landers 2016); as a consequence, this piece of slop is not a trifle, but an intervention with political ramifications within and outside of the US, one with the potential to ‘recast’ public diplomacy and the ‘subjects, objects, and relationships that constitute the international system in real-time’ (Lerner and O'Loughlin 2023, 2).

The video is also, and please excuse the pun here, a *shitpost* in both in the figurative and literal sense: a social media post designed to be ‘deliberately offensive and/or non-sensical’, shared mainly for the ‘lulz’ (Baspehlivan 2024, 53). Based on the low-quality graphics and spatial mismatches (Trump’s nose remains outside the oxygen mask while it strangely adheres to the lower third of his face), the short film exhibits the hallmarks of being cheaply made using an off-the-shelf, free-to-use visual generative AI tool. Like similar politically-inflected shitposts, it has been published on social media in order to simultaneously entertain, rally, troll, and/or enrage its (un)targeted audience. Rather than simply circulating on alt-right forums, the ‘King Trump’ video prompted news coverage in multiple major outlets in the US and around the globe, thus joining a host of other Trump-reposted AI-generated videos, memes, and altered images making news in their own right. Prominent examples include Trump as the successor to Pope Francis, the remaking of the war-torn Gaza Strip into a ‘Trump Gaza’ seafront riviera, an image of Trump surveying an ostensibly annexed Canada, the fictive FBI ‘arrest’ of Barack Obama in the Oval Office, and Trump’s 2024 Democratic opponent Kamala Harris addressing a packed stadium under the banner of the USSR (see Thompson 2025).

Are these AI-generated humorous images important for global politics and public diplomacy? We argue the answer is a resounding ‘Yes’. Moreover, we contend that those in the field who work with the concept of strategic narrative are likely to agree. The current state of affairs is particularly problematic given the instability that such ambiguous and incoherent signalling brings to the ‘dual role’ that strategic narratives play in ‘plotting political action and reshaping international imaginaries’ (Lerner and O'Loughlin 2023, 4). The global circulation of such videos and the attendant international ‘media echo’ (Stacey et al. 2025, 5) of their content (and meaning) suggest they have an impact and audience that overspills the domestic sphere. Through social media, Trump has become the ‘face of the state’, i.e. the dominant interlocutor of America’s strategic narrative; and with videos such as these, his ability to shape ‘how laypeople understand world politics’ is increasing at varied scales around the globe (Ku 2025, 2–4). In this article, we interrogate recent examples of Trumpian AI videos as a point of departure for reflecting on the significance of public diplomacy and strategic narratives in the age of generative artificial intelligence.

Trump—as a part of a raft of neo-authoritarian strongmen (Vladimir Putin, Recep Tayyip Erdoğan, Rodrigo Duterte, Jair Bolsonaro, Viktor Orbán)—is redefining what public diplomacy means in an era defined by geopolitical performativity (Terry and Makarychev 2022), diplomatic trolling (Dylan and Colley 2025), and ‘hybrid warfare’ in which the ‘domination of the information space [is] as important as any kinetic movement of personnel or deployment of firepower’ (Cull 2016, 244). What these leaders say locally has global ramifications and shapes how their nations and other countries are viewed globally.

Consequently, the projection of American power via Trump as a shitposting Strong Man, despite its seeming ‘ideological vacuity’, has meaning that transcends simply ‘owning the Libs’ (Hartley 2023, 69). Rather than an ideological regime, Trump pursues a ‘dramaturgical regime’ which builds traction around certain ideas, representations, and concepts that gains ‘traction through repetition and gradual normalisation’ (Stacey et al. 2025, 4). Despite the apparent lulz-based ‘entertainment value’ of the video, this—and similar—videos serve as a vehicle of ‘official’ White House policy, challenging international audiences to make sense of the meaning (in this case, the shit-bombing of activists observing their First Amendment right to peaceful protest against an aspirant monarch).

Social media content, when shared by sitting presidents, shapes how those countries are perceived, presenting the state as a ‘unitary actor’, that is a coherent whole operating in the international system (Ku 2025, 2). However, there is a difference here between Orbán sharing a photo on Instagram of himself in ‘military, sports, and food’-based setting to enhance his personal embodiment of traditional ‘Hungarian masculinity’ (Szebeni and Salojärvi 2022, 821) or Bolsonaro’s use of Facebook to lambaste ‘mainstream media outlets, the government, and his adversaries’ (Machado et al. 2021, 45). In these instances, we see carefully curated (re)presentations of the leader as the state embodied.

Conversely, Trump’s crowd-sourced videos are symptomatic of how genAI is now shaping the global media ecology in a way that the ‘the associated material, knowledge, data, logistical, labour, and political, cultural, economic, and ideological infrastructures behind [AI]… functions as an empire’ that is ‘deeply steeped in heteropatriarchy, racial capitalism, white supremacy, and coloniality’; in short, the continuation of ‘empire’ in the AI realm is not a ‘glitch’, it is a feature (Tacheva and Ramasubramanian 2023, 1–2). Alongside this, as is apparent in Trump’s regular sharing of genAI content (memes, deep fakes, mash-ups), AI is emerging as the preferred and increasingly emblematic ‘aesthetic form for the far right’ (Watkins 2025). So today both the structure and the aesthetics of our current digital media environment—on which the bulk of public diplomacy plays out—are shaped by the rise of genAI and the slop it pumps out. As a result, over ten years on from the publication of the foundational book by Miskimmon et al. (2013), we suggest that the concept remains useful, but caution that if it is to maintain its promise for understanding how (geo)political actors shape order through narrative projection, those of us who work with the concept need to account for the rise of what we call the *resonant slop machine*.

Considering the centrality of the rise of a ‘new media ecology’ to Miskimmon et al.’s original contribution, the integration of AI into many facets of everyday online life represents a sea change worth considering. Novel recent developments include the introduction and widespread adoption of generative AI (think ChatGPT serving as a now ubiquitous crutch for nearly any task on- or offline), sophisticated software programmes that can produce highly believable ‘deep fakes’ (consider a fabricated Volodymyr Zelenskyy telling his nation’s defenders to lay down their arms at the start of Russia’s full-scale invasion of Ukraine), politicised AI hallucinations (remember X’s AI Grok refashioning itself as ‘MechaHitler’), and the ubiquity of algorithmic personalisation in a post-truth information environment (with ‘Stop the Steal’ metastasising into the 6 January 2021 assault on the US Capitol as a key example). Subsequently, it is time to revisit the principles of strategic narratives in order to understand how the current wave of rapidly proliferating ‘digital technologies are producing a qualitative sense of change *and* observable changes in practices in all spheres of life’ (Miskimmon et al. 2013, 11) replete with disruptions to the ‘existing balance’ of relations that affect ‘authority, legitimacy, and—ultimately—power’ (Miskimmon, O’Loughlin, and Roselle 2017, 9).

We begin by briefly reflecting on our respective employment of strategic narrative as a way of comprehending global politics and public diplomacy. We then return to the Trump shitpost as a techne that reveals several important dynamics of contemporary global (geo)politics in the chaotic *new* new-media-ecosystem abetted by AI, employing techniques drawn from *Strategic Narratives*, specifically the *behaviour* of the actor, how the setting is *staged*, the *framing* of the action, and finally the *meaning* of the (proposed) resolution. We then turn to the work of Connolly (2005) to theorise the resonant slop machine and the global political-socio-cultural-economic-technological realm it condemns us to: an age that is fractured (Rodgers 2011), a zone that is flooded (Starr 2020), and world (politics) that is enshittified (Doctorow 2025).

In the era of the resonant slop machine—characterised by AI run amok, cascading climate chaos, unpunished genocide, unpredictable hybrid warfare, skyrocketing wealth inequality, unhinged nuclear brinkmanship, and the transnational resurgence and solidification of the global far-right—public diplomacy is increasingly polluted by populism, theatricality, and stunts. Consider the geopolitical implications of Trump’s ‘coercive trolling’ (Dylan and Colley 2025, 10) during his televised attack on Zelenskyy’s sartorial choices (not wearing a suit), aided by his ‘Thank you’-demanding vice president JD Vance who later claimed the Oval Office argument to be ‘useful’ in elucidating ‘real issues of disagreement between the United States side and the Ukrainian side’ (Starcevic 2025). In this new milieu, very little seems strategic, and narratives often seem to not make sense. What then is to be done? We close with tentative suggestions on ways forward for those of us interested in working with, analysing, and perhaps producing strategic narratives to prompt more effective public diplomacy and a better world politics for all.

## Strategy, narrative, scholarship

Considering that Miskimmon et al. drew on *A Grammar of Motives*, Kenneth Burke’s (1992 [1945]) classic study of the dramatistic pentad of Act, Scene, Agent, Agency, and Purpose, we call attention to the current era of power projection via the notion of what might be called geopolitical dramaturgy, which when interspliced with public diplomacy and strategic narratives, helps us better understand the resonance of AI slop: how it works, why political actors use it, and what goals it might achieve. But before proceeding, we both provide a précis on our own orientation towards strategic narratives and how we have employed the concept in our work—foundations that subsequently inform our further reflections.

### Positioning strategic narrative in our work: part I—Crilley

It is not an overstatement to state that early work on the concept of strategic narratives, and in particular Miskimmon, O’Loughlin, and Roselle’s *Strategic Narratives: Communication Power and the New World Order* (2013) changed my life. As a first year PhD student interested in understanding the role of images of conflict on social media, it proved to be an essential read for helping me ground ideas, develop methods, and work on my own contribution to knowledge. The fundamentals of strategic narratives are clear: 1) narratives ‘shape our world’ and the behaviours of those in it; 2) narratives are used strategically by political actors; and 3) the media and communication ecology ‘affects how narratives are communicated and flow, and with what effects’ (Miskimmon et al. 2013, 1). The rest of the book engages with a rich body of literature, offers a detailed conceptualisation and theorisation of strategic narratives, and develops a convincing argument throughout about how and why strategic narratives matter in global politics. Over a decade on, much of the book (covered in my own highlights, annotations, and multiple !!! in the margins) still holds up, and it is no surprise that many people working in IR, and in particular in the field of public diplomacy, have taken the concept and ran with it in their own work.

Indeed, my first ever published academic article, ‘Seeing Strategic Narratives?’ (Crilley 2015), was a short reflection on visuality and strategic narratives, and since then I have forged a career by working with and around the concept. This work has largely been collaborative and has involved developing ideas around strategic narratives and how they are interlinked with visuality and visual media, including how political actors produce, circulate, and make sense of visual narratives of global politics (Crilley et al. 2020a, b). In addition to this, I have worked with colleagues to draw attention to how strategic narratives are bound up with emotions (Crilley and Chatterje-Doody 2020), whilst also highlighting how important the site of the audience is in understanding who the consumers of strategic narratives are, and how they actually make sense of them (Crilley et al. 2020a, b, 2022a; Hutchings et al. 2024). In recent years, I’ve gone ‘nuclear’—and become interested in how strategic narratives, public diplomacy, and a changing media ecology shape the politics of nuclear weapons, arguably one of the most pressing issues in contemporary international relations (see Crilley 2024, 2025).

### Positioning strategic narrative in our work: part II—Saunders

Coming out of my interdisciplinary PhD in Global Governance, I struggled to find the appropriate set of literatures to help me properly understand, analyse, and critique a new problem that I was grappling with: How and why the Republic of Kazakhstan had effectively declared war on the fictional character of Borat (portrayed by the British comedian Sacha Baron Cohen)? (see Saunders 2007, 2008a, b). I eventually gravitated towards the field of nation branding to fill in the lacunae that the literatures of International Relations and critical geopolitics revealed to me, with the journal *Place Branding and Public Diplomacy* becoming a mainstay of my reading via mind-broadening essays by Anholt (2006), Andersson (2007), Szondi (2007), and Hospers (2009). Yet, a yawning disconnect continued to linger, which I tried to fill leaning on the much-underappreciated work of Kunczik (1997) and Rusciano and Ebo (1998), as well as other scholars whose work on images as tools of statecraft rhymed with my approach. Enter *Strategic Narratives* (2013): with this text, the post-structuralist inside me had found its North Star. Since my first perusal of the book, the notion that power in the world order is performance has been at centre of my scholarship (though I think it already was there, though in an incipient and poorly expressed form).

My most direct engagement with the work of strategic narratives came in the form of my essay ‘Popular Geopolitics, Strategic Narratives, and Soft Power in *Viking* (2016) and *Guardians* (2017)’, which as was published in *Cinema and Soft Power: Configuring the National and Transnational in Geo-Politics* (Dennison and Dwyer 2021). In this chapter, I adapted the analytical lenses of Miskimmon et al. (which in turn were adaptations of Burke’s dramatological ‘grammar’) to interrogate how state-aligned filmmakers in Russia used these blockbuster films as a tool of soft power and public diplomacy. I found that by trading in a productive ‘feedback loop’ of tropes, visual rhetoric, and storytelling structures, the cultural producers, both with strong ties to the Kremlin, crafted works of art that were intelligible to a global audience, while advancing resonant strategic narratives associated with (post-)Soviet Russia, therein presenting one narrative at home and another abroad (Saunders 2021, 161). Either explicitly or implicitly, I have returned to the well of strategic narrative to assess the screening of televisual diplomacy in the DR/Netflix documentary on Rufus Gifford *I am the Ambassador/Jeg er ambassadøren fra Amerika* (2014–2016) (Saunders and Vessels 2019), how Norwegian national identity and state value systems manifest in the thrillers *Occupied* and *Nobel* (Saunders 2020), and as a mechanism for explaining Ukraine’s repositioning of its place in the geopolitical congeries after the Euromaidan Revolution (Saunders 2025). And the impact of the work of Miskimmon et al. work is not simply scholarly, as I assign their *Media, War & Conflict* article ‘Strategic Narrative: A New Means to Understand Soft Power’ (2014) in my Comparative Foreign Policy course, viewing it as a most eloquent example of explaining to students how public diplomacy actually works.

Together then, we have both found strategic narratives to be a vital contribution that has informed our work to date. Our shared interest in strategic narratives has led us to build upon the early work by encouraging an approach to strategic narratives and public diplomacy that is attuned to the role of visual media, emotions, affect, humour, and pop culture. These strands of interest come together and are pertinent in making sense of our current media ecology and the future of strategic narratives and public diplomacy in the context of the resonant slop machine.

## No clear strategy, few clear narratives: enter the resonant slop machine

Donald J. Trump has built his brand—as an entrepreneur, celebrity, and politician – through the efficacious employment of visuality, spectacle, and by manipulating elements of a changing media ecology. From professional stunts to garner newspaper coverage, to his cameos in films, television series, and World Wrestling Federation events, the Trump of the 1980s and 1990s primed the pump for *The Apprentice* (2004–2015), which in turn set the stage for Trump the Presidential Candidate (Gabriel et al. 2018). After being barred from the dominant social platforms after the Capitol Riots, he established his own social media platform: Truth Social. With its alt-right orientation, the new medium established an environment for the jungle of AI-inflected (geo)political slurry discussed above to flourish, providing nothing less than hothouse-like conditions for the ‘erosion of knowledge’ in favour of ‘slopaganda’ (Gross and Colson 2025, 291–292).

Indeed, the ‘dominant symbolic order’ (Stacey et al. 2025, 1) of global politics is under threat from an epistemic regime where there are no more accepted facts, figures, authorities, or experts to produce consensus: one’s sides ‘truth’ is the other side’s ‘lies’. In this era of AI slop, political actors produce ‘strategic ontologies’ (Lerner and O'Loughlin 2023, 2), competing to project coherent narratives and to convince others that their preferred version of reality is the-world-as-it-is. When actors employ such genAI videos (which are often clearly unreal) as part of their means of depicting that world then the very landscape of ‘self-presentation and impression management’ takes on surreal quality (Ku 2025, 2). Through the rise of AI, the already existing ‘multiple and conflicting mediated realities’ (Manor 2024, s49) brought about by the rise of personalised social media, public diplomacy, disinformation, and digital propaganda become even more untethered from a common ground of knowledge, epistemic authority, and agreement around facts and expertise. This instability is heightened by tactical efforts to ‘blur the boundary between actors and audience’ (Stacey et al. 2025, 9), with Trump platforming crowdsourced AI slop as if it were a report from a respected think tank or a State Department communiqué. When questioned about Trump’s response to the ‘No Kings’ protests, Republican Speaker of the House Mike Johnson responded to the furore over the ‘poop bombs’ by saying that Donald Trump ranks as the ‘most effective to ever use social media’ (Garcia 2025), while the White House assistant press secretary Liz Huston declared: ‘No leader has used social media to communicate directly with the American people more creatively and effectively than President Trump’ (Thompson 2025). Effective, perhaps, but is it strategic? Does it coalesce into a coherent narrative? And does that even matter anymore?

Returning to the kingly-fighter pilot shitpost, we want to examine the video as tool of both geopolitical dramaturgy and meaning-making in the context of the formation, projection, and reception of an American strategic narrative. Narrative is constructed through the building-blocks of actors, events, plot, time, setting, and space (Miskimmon et al. 2013, 5). Our actors are King Trump as the protagonist, with the ‘No Kings’ protestors as the antagonist. Positioned as latter-day Lieutenant ‘Maverick’ (the hotshot naval pilot played by Tom Cruise), Trump thus embodies a globally recognisable figure (given the success of the original *Top Gun* and even more so its 2022 sequel *Top Gun: Maverick*) that provokes nostalgic resonance through its 1980s hard-bodied form of masculinity (Jeffords 1993), a reprise of other similar meme-culture performances including Trump as Rambo and Rocky (Sylvester Stallone), as well as Conan the Barbarian (Arnold Schwarzenegger) and even *Wall Street* corporate raider Gordon Gekko (Michael Douglas). By extension, the protestors are the faceless enemy (read as the Soviets in *Top Gun*), which are up to no good, and are quickly dispatched with the weapons at hand (in this case, raw sewage), thus giving us the events. The plot is simple, but meaningful and gendered. America, as a freedom-loving land of *men* of action, is under threat and will respond. The visual and narratological appeal to the 1980s, the last time when there was a clear (albeit American-made) demarcation between ‘good guys’ and ‘bad guys’, resonates with global publics that see post-millennial politics (domestic and international) as senseless. Personified via the rule-breaking ‘strong man’ of action (the Mavericks of the world), Reagan-era ideological certainty, whatever its faults, made simple sense.

Yet, Trump as King complicates the diegesis, introducing an element of dissonance into the national narrative. The setting, rather than a dogfight in the sky, is an American city, its streets filled with activists rallying against the once-sacrosanct value of all US citizens: a complete and total rejection of monarchy (see Brewster 2025). The time is now, an America where Trump has attacked Democratic-governed cities as ‘crime-infested rat holes’, ‘war ravaged’, and ‘killing fields’, raising the prospect of employing the Insurrection Act if they ‘don’t learn their lesson’ and telling his own military that these urban spaces should be used as ‘training grounds’. Treating the space of the narrative holistically, we view King Trump’s AI-generated *Top Gun* shitpost as operating within a geopolitical imaginary of an authoritarian America, where MAGA ideology—as incoherent and protean as it may be—trumps everything else. In this environment, ideology has become ‘untethered to a robust and defensible epistemic system’, therein making it ‘free to operate only on emotions, and the worst of emotions seems to be the most politically profitable—fear, anger, and vindication’ (Hartley 2023, 3). As loyalty to the leader and ‘owning the Libs’ (i.e. making Trump’s opponents look stupid by triggering them into overreaction or denying them an opportunity to debate by failing to play on a level epistemic field) has come to matter more than democracy and America’s destiny.

While we must remember that neither Trump nor his administration made this video, its circulation by the President is a discursive act, and one which impacts the ‘set of meanings and practices about what is say-able and know-able’ in the world and important in establishing particular ‘roles which actors fill’ (Miskimmon et al. 2013, 7). As part of a larger assemblage of the use of humour as a ‘political technology’ among right-wing actors across the globe, often breaching ‘established diplomatic registers’ associated with serious issues (Hemment 2022, 201–202), this satirical baiting of the American Left via the AI slop machine is a risky feint. By employing elements of the country’s robust popular-geopolitical repertoire, wherein militarised masculinity is glorified through the deployment heavy firepower (see Salter 2014), this scatological spectacle can easily be misunderstood as an innovation on the country’s strategic narrative, suggesting—through metaphor—that Trump is ready and willing to strike against his own population. Combining the high-octane soundscapes of ‘Danger Zone’ and the aesthetics of Maverick’s martial jingoism, the representation of Trump as a militarised monarch makes meaning in the current media ecosystem, one which is more globally connected, visually attuned, and (potentially) more resonant than ever before.

## Theorising the resonant slop machine

Drawing on Peters’ *The Marvelous Clouds* (2015), the Introduction to *Forging the World: Strategic Narratives and International Relations* reminds us that ‘Media are civilisation ordering devices’ (qtd. in Miskimmon, O'Loughlin, and Roselle 2017, 10). So then how do we, as critical scholars of International Relations, make sense of strategic narratives and public diplomacy, in an age of geopolitical dramaturgy where AI is ‘vomit[ing] out stitched-together stock footage with robotic voice-overs’ (Madsen and Puyt 2025)? Should we just accept that which is offered up to us as a ‘degraded form of digital nutrition, consumed more out of necessity than intrinsic value’ (Gross and Colson 2025, 290)? We answer in the negative. Instead, we turn to the work of Connolly (2005), who, writing at the apex of the George W Bush’s intervention in Iraq, unveiled what was all around us: the evangelical-capitalist resonance machine. Reflecting on the interconnectedness the right, capitalism, and evangelicalism, he noted that there were few clear narratives shaping American and global politics, instead there was a set of ‘energised complexities of mutual imbrication and interinvolvement, in which heretofore unconnected or loosely associated elements *fold, bend, blend, emulsify, and dissolve into each other*, forging a qualitative assemblage resistant to classical models of explanation’ (2005, 870).

Fast forward to today and running this through what Page and Dittmer label the ‘white-male dissonance machine’ of Trump and MAGA (geo)politics, we see the body of the President as ‘a central, performative element allowing this assemblage to cohere’ (Page and Dittmer 2016, 77). Bits and bobs of America’s strategic narrative are sucked into the eddies and whirlpools of production from which the Trumpian visual AI slop emerges, thus guaranteeing, at least for some, resonance—a modus of relating to the world that avoids alienation (Rosa 2019)—across various (battle)fields of power. With regards to media, Dittmer and Bos note that media resonance occurs when ‘synchronicity between two or more affects’ produce a more powerful affect than they would produce individually, thus reinforcing each other ‘like tuning forks vibrating at the same frequency’ (2019, 121). Furthermore, Holland points us to the concept of geopolitical resonances, which he defines as the ‘ways in which popular media can determine events and outcomes in global politics’ (2022, 125), to demonstrate the centrality of media and pop cultural artefacts, even fatuous ones, in contemporary power relations across and within states.

We argue that Trump’s (geo)political dramaturgy, especially via flooding the zone with outrageous output drawn from the resonant slop machine is exacerbating trends of fracture across the American polity *and* the international community of nations, therein resulting as a next-level enshittification of (global) politics. With regards to the realm of public diplomacy, genAI is handmaiden to ‘nefarious actors’ as they seek to profit from irreality, helping them foment differences between realities that can no longer be reconciled in an environment built on deterritorialised digitality, not interpersonal engagement (Manor 2024, s50). This ‘accelerating decay of things that matter to us’ (Doctorow 2025: 1) has inflicted the digital platforms and media that underpin our daily lives, and given how these platforms and media are central to global politics, this cascading decline now bleeds into the actors and institutions that govern and shape global politics as the quality of public diplomacy appears to hit rock bottom.

Rather than positing that Trump and his MAGA assemblage are somehow unique in their theatrical enshittification of (geo)politics, we want to briefly engage with the Russian concept of *stiob*, which, according to Hemment, has been operationalised by Sergei Lavrov and other Russian officials with some regularity over the past decade. Adapting Soviet-era practice of mimicking authoritative political communication with such discursive accuracy and performative sincerity that it becomes indecipherable from actual policy statements, Russian diplomats have made use of ‘ambiguous play and satirical borrowings via the circulation of memes and other political theatrics’ to disrupt the norms of the international statecraft, particularly in Moscow’s dealings with Washington (Hemment 2022, 201). As Saunders (2019) has argued elsewhere, Putin—via the medium of RT—effectively produced a satirical ‘supervillain’ version of himself for foreign consumption via highly-performative geopolitical stunts, which in turn were consumed and repurposed in right-wing media circles in the US to feminise the sitting US president, Barack Obama. Similarly, RT’s utilisation of humorous, shitpost-style videos produced by influencers (see Crilley and Chatterje-Doody 2021; Saunders et al. 2022) also points towards a broader global strategic politics of humour that has emerged and laid the foundations for today’s AI generated resonant slop machine.

Over-the-top geopolitical theatricality which taps various strains of their respective national narratives has also occurred via the discursive and bodily performances of Boris Johnson (UK) and Silvio Berlusconi (Italy) (see Hemment 2022). This trend of style-over-substance wherein public diplomacy goes off the rails reached a peak with the ‘Wrestlemania’-like optics, framing, and staging of the Trump-Kim summit wherein MAGA-specific narrative structures of ‘highly personalised contest’ between ‘hero’ and ‘heel’ drawn from the realm of professional wrestling guided American foreign policy towards a short-lived and ultimately hollow rapprochement with Pyongyang (Day and Wedderburn 2022, 8). This movement from clear, credible strategic narratives deployed professionally as tools of smart public diplomacy towards factually misleading, satirically regressive, seductively interactive, and ultimately ephemeral visual media grounded in meme culture has reached warp speed with the widespread adoption of AI. We call this the resonant slop machine.

For Connolly writing in 2005, global politics and America’s role in it was explained through his theorisation of what he referred to as the capitalist-evangelical resonance machine: an assemblage with an ‘economic leg’ and a ‘religious leg’ (Connolly 2005, 876) whereby American evangelical Christianity and neoliberal capitalism, buoyed by the Republican party and conservative media, mutually amplified one another despite core divergences of identities and sensibilities. Here, ‘affective intensities’ (Connolly 2005, 880) and a ‘politics of existential revenge’ (Connolly 2005, 881) were central to a feedback loop of inequalities caused by capitalism and an evangelical worldview that legitimated such inequalities. As synchronised economic, spiritual, media, and political forces shaped by perceptions and inflected economic interests intensified solidarities between these communities and contributed to the hegemonic dominance of the right in American politics and society.

Today, Connolly’s ideas warrant revisiting. Given the changing media ecology’s enabling of the emergence of a diverse global right, taking its cues from the American alt-right, but also with worldwide reach (as well as regional, national, and local nuances), we see an ideological assemblage that has moved beyond the binary relation between evangelical and capitalist movements which itself was largely the product of the broadcast news media environment of the early noughties (see Abrahamsen et al. 2024). Now, the largely one-to-many media ecology has given way to the rise of social media and a Web 2.0 many-to-many media ecology (Facebook for example was made available to the public in September 2006—a year after Connolly’s article was published) that is currently in the early days of AI integration—through AI generated content, chat bots, AI centric platforms, and more. In addition, the American and global right are now more diverse than the cowboy capitalist, neocon hawkish, evangelically grounded right of the 1990s and early 2000s.

Enter the resonant slop machine: a global political-socio-cultural-economic-technological assemblage or ecosystem whereby AI generated slop ‘infiltrates perception and inflects economic interest’ (Connolly 2005, 871) as novel and slippery affinities of identity energising global politics in ways that outstrip the corporate greed and religious fever of Connolly’s evangelical-capitalist resonance machine. When placed on offer, these affinities proved attractive to political plethora which Hillary Clinton infamously referred to as ‘basket of deplorables’, a broad umbrella of the (coming) MAGA movement incorporating a Republican party remade in Trump’s image, of billionaire kleptocrats, anti-state libertarians, Second Amendment diehards, Christian nationalists, antisemites, white supremacists, Zionists, xenophobes, misogynists, incels, tech bros, trad wives, the supposedly left-behind working class, free-speech purists, and conspiracy theorists (cf. Page 2021; Weichelt 2021; Feagin and Ducey 2025; Wolf et al. 2025; Agnew 2025).

As before, the groups do not share the same—in Connolly’s terms—identities and doctrines, but are connected through ‘affinities of sensibility’ (2005, 871) and are, in his words again, an ‘aggressive constellation’ (2005, 871–872). As with the earlier evangelical-capitalist resonance machine, today’s resonant slop machine grants a constellation of groups ‘a spiritual disposition to existence’ whereby ‘their ruthlessness, ideological extremism, readiness to defend a market ideology in the face of significant evidence, and compulsion to create or condone scandals against any party who opposes their vision of the world, express a fundamental disposition toward being in the world’ (Connolly 2005, 872).

Together in grievance, anger, and (gendered, racialised) thymos (Ganesh 2020), the resonant slop machine produces the connective tissue that brings ‘parties together amidst variations in religious doctrine, economic creed, and life conditions’ and ‘any constituency or social movement that crosses them is subject to sharp castigation and accusation’ (Connolly 2005, 872). Today, this is partly taking the form of AI generated shitposts but is also apparent in broader attacks on civil liberties, targeted attacks on free speech, and the creation of conditions where political violence is encouraged and condoned.

Now, as with Connolly’s resonance machine, core to the movement is the mantle of victimhood (2005, 873), one which is worn proudly *in* or *out* of power despite the growing resonance of right-wing discourses in most aspects of American (and increasingly global) society (see Eroukhmanoff 2025). The resonant slop machine manifests in affective intensities, grievances, and polarisation driven and amplified by algorithms operating across platforms owned by billionaire oligarchs who pay fealty to Trump (and his ilk). Generative AI lowers the entry costs not only for media consumption but also production, with political figures like Trump and Vance using mimicry and memes to show that they are in on the joke. They too are doing it for the lulz, and whilst everyone knows that Trump is not literally flying a fighter jet and dropping shit on his opponents, the spectacle nonetheless has a return on investment as the resonant slop machine enables a distinct dumbing down of political communication and a literal depiction of the very simple message that Trump shits on his opponents. Can strategic narratives save us in such an ontologically decimated milieu?

## Salvaging strategic narratives and public diplomacy in the era of AI slop

In this paper we have begun to theorise the resonant slop machine and its impact on global politics, especially in regard to the scholarship of geopolitical dramaturgy, strategic narrative, and public diplomacy. Now, what do we do? Beyond being researchers attuned to the elemental aspects of the resonant slop machine—visuality, humour, emotion and affect, the rapidly evolving media ecology, and the interconnectedness of awfulness driven by political economy of aggrieved identity—we should take heed of Connolly’s earlier exploration of what to do in response to the resonance machine. First, know that the resonant slop machine is a threat to public diplomacy as much as it is to democracy, and that it cannot be approached, assessed, or ‘interrogated in a mood of political neutrality’ (Connolly 2005, 870). When heads of state abandon the dutiful management of strategic narrative in favour of lulz wrought from mash-up content drawn from the ‘primordial soup’ of artificially generated content in the digital realm (Gross and Colson 2025, 289), they are accelerating the ‘atomisation of reality’ at home and abroad (Manor 2024, s50). Strife, polarisation, demhumanisation and discord will make meaningful engagement less possible over time as what was once common ground becomes a morass of low quality digital clutter, half-baked memes, and alienating provocations dressed up as genuine efforts at managing international relations.

In an age of AI slop, where political actors pivot from one genAI meme or video to the other and communicate with publics in ways that seem devoid of strategy, narrative structure, or diplomatic purpose, we must appraise what we have taken for granted in the study of strategic narratives and public diplomacy. The resonant slop machine, understood as an ‘epistemicidal’ innovation that ‘complicates knowledge, erodes trust in online media, and reconfigures our aesthetic expectations’ (Gross and Colson 2025, 291–292) could signal the end of public diplomacy, as communication descends into a media ecology of madness where shitposting becomes the norm, not the exception. At a time of the resurgent right that is riding the resonant slop machine to an unknown but overtly oppressive future for the planet and the majority of people on it, perhaps strategic narratives will not help us in our efforts for progress? Instead, perhaps we should embrace joviality, the lulz, and a fractured politics of the silly and absurd (see Baspehlivan and Wedderburn 2024), all with the goal of making (public) diplomacy great again.

## Data Availability

No datasets were generated or analysed during the current study.
